# Evaluation of the Effectiveness of Single-Nucleotide Polymorphisms Versus Microsatellites for Parentage Verification in Horse Breeds

**DOI:** 10.3390/vetsci12090890

**Published:** 2025-09-15

**Authors:** Dongsoo Kim, Sunyoung Lee, Baatartsogt Oyungerel, Giljae Cho

**Affiliations:** 1 College of Veterinary Medicine, Kyungpook National University, Daegu 41566, Republic of Korea; dsfarriery@hanmail.net; 2 Racing Laboratory, Korea Racing Authority, Gwacheon 13822, Republic of Korea; yeji0628@kra.co.kr; 3 School of Animal Science and Biotechnology, Mongolian University of Life Sciences, Ulaanbaatar 17024, Mongolia; baatarsogt@muls.edu.mn

**Keywords:** horse, genotyping, parentage, SNP markers, STR markers

## Abstract

This study aimed to generate information for parentage testing in horse breeds using microsatellites (STRs) and single-nucleotide polymorphisms (SNPs). Genotype data were obtained from 189 horse hair root samples, including 38 Thoroughbreds (TBs), 17 Jeju horses (JHs), 20 Quarter horses (QHs), 21 American Miniatures (AMs), and 93 Mongolian horses (MHs), using 15 STR markers and 71 SNP markers. Comparative analysis revealed that the mean expected heterozygosity ranged from 0.468 (AM) to 0.491 (JH) for SNPs and from 0.695 (TB) to 0.791 (MH) for STRs. The mean observed heterozygosity ranged from 0.415 (AM) to 0.487 (MH) for SNPs and from 0.706 (JH) to 0.776 (MH) for STRs. The mean polymorphic information content ranged from 0.349 (AM) to 0.364 (MH) for SNPs and from 0.635 (TB) to 0.761 (MH) for STRs. The inbreeding coefficient ranged from −0.009 (MH) to 0.113 (AM) for SNPs and from −0.058 (TB) to 0.043 (AM) for STRs. The cumulative exclusion probability (PE) for the 71-SNP panel exceeded 0.9999, indicating that SNP markers may be sufficient for parentage testing. In comparison, the STR markers yielded a combined PE of 0.9988 when one parent was known and 0.9999 when both parents were known. These findings highlight the potential of SNPs as alternatives to STRs for routine paternity verification in horses.

## Simple Summary

The International Society for Animal Genetics is currently investigating the use of single-nucleotide polymorphism (SNP) markers as an alternative to short tandem repeat (STR) markers for equine parentage verification. This study, which examined five horse breeds, confirms the potential of SNP markers to replace STR markers in routine paternity testing.

## 1. Introduction

Horse registration primarily relies on evaluating physical characteristics and genetic traits. Genetic traits are analyzed according to Mendel’s laws of inheritance using blood or DNA typing. Currently, DNA typing in horses is based on two types of genetic markers: microsatellites, or short tandem repeats (STRs), and single-nucleotide polymorphisms (SNPs).

For nearly 40 years, blood typing was the standard for parentage verification in horse breed registration. However, owing to its limitations, it was replaced by STR analysis in the early 2000s. STRs can be analyzed from blood and hair follicles (hair roots), and the process is largely automated using specialized equipment, offering an accuracy rate of 99.99% [1,2]. Recently, research in several countries has focused on introducing SNP markers as a potential alternative to STR markers in horse registration [3,4,5,6].

SNPs are a novel type of genetic marker that offer several advantages for evaluating origin, evolution and genetic diversity across various species, including animals [3,4,7], fish [8], and humans [9]. Inaccurate pedigree records and individual misidentification can compromise the accuracy of genetic evaluations, ultimately undermining the efficiency of breeding programs. Identifying discriminative SNP markers offers a valuable opportunity to leverage genomic data effectively, for example, in determining the population of origin of unidentified individuals. Numerous studies have investigated discriminative SNP markers and genetic diversity extensively [3,4,7]. These markers can be used to develop cost-effective, customized panels for breed identification and offer a reliable solution for the tracing of breed-specific branded products.

In this study, we assessed the possibility of replacing the STR marker-based analysis currently used for paternity testing and individual identification in horses with SNP-based analysis. To provide supporting evidence, we applied SNP analysis to actual cases of horse paternity testing.

## 2. Materials and Methods

### 2.1. Sample Collection and Genomic DNA Extraction

Genomic DNA was extracted from 189 horse hair root samples including 38 Thoroughbreds (TBs), 17 Jeju horses (JHs), 20 Quarter horses (QHs), 21 American Miniatures (AMs), and 93 Mongolian horses (MHs) using the automated Nimbus KingFisher Presto system (Hamilton/ThermoFisher Scientific, Waltham, MA, USA), following the manufacturer’s protocol [10].

The extracted DNA was quantified using a NanoDrop 2000c spectrophotometer (Hamilton/ThermoFisher Scientific, Waltham, MA, USA).

### 2.2. STR and SNP Analysis

A panel of 15 microsatellite loci (AHT4, AHT5, ASB2, ASB17, ASB23, CA425, HMS1, HMS2, HMS3, HMS6, HMS7, HTG4, HTG10, LEX3, and VHL20) was used for the genetic analysis of Equidae. This panel is routinely used for parentage verification in the Korean Thoroughbred Horse Registration system. Polymerase chain reaction (PCR) amplification was performed according to the manufacturer’s instructions (Equine Genotypes Panel 1.1 Kit, ThermoFisher Scientific, USA) and the method described by Dimsoski [11].

PCR products were analyzed using an automatic genetic analyzer (AB 3500XL Genetic Analyzer, ThermoFisher Scientific, USA). Electrophoresis was conducted on POP 7 polymer (ThermoFisher Scientific, USA) at 15 kV, and data were processed using Data Collection (ver. 5.0) and Gene Mapper (ver. 5.0) software (ThermoFisher Scientific, USA). Allele discrimination was performed according to the guidelines of the International Society for Animal Genetics (ISAG) Equine Genetics and Thoroughbred Parentage Testing Standardization Committee. Alleles were represented using alphabetical symbols, arranged from smallest to largest, with the mid-sized allele designated as “M”. STR genotyping followed the procedures described by Kakoi et al. [12] and Tozaki et al. [1], with minor modifications to HTG10.

**Figure 1:**
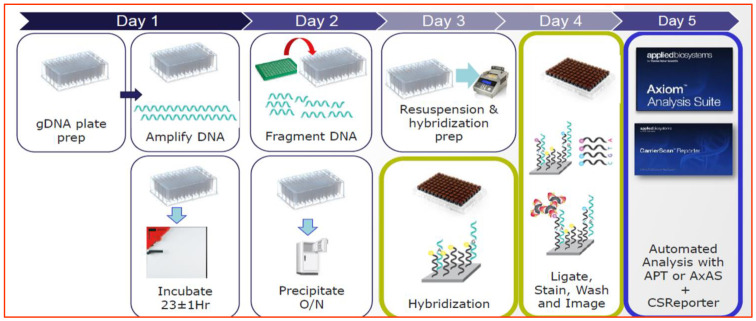
Workflow of single-nucleotide polymorphisms analysis using ThermoFisher equipment.

SNP analysis was conducted using the Axiom Equine 670K array, which is relatively accurate among the SNP arrays currently being studied in horses (GGP Equine V5, Equine 80 Select, Axiom Equine 670K array, GBS). A total of 71 SNPs were analyzed. These markers are included in the Core Panel of 381 autosomal SNPs currently under study by ISAG [13,14]. The workflow for SNP analysis is illustrated in Figure 1. Before analyzing the SNPs, quality control (QC) and quality assurance of genotype data were performed according to the method reported by Lee et al. [15].

SNP markers underwent a four-stage pretreatment process: DNA amplification, fragmentation, resuspension, and hybridization, followed by ligation, washing, and image scanning.

Final genotyping was performed using the Axiom Equine Genotyping Assay (Axiom™ MNEc670, ThermoFisher Scientific GeneTitan MC Instrument, USA) [15,16].

### 2.3. Genetic Diversity Analysis

Allele frequencies for each locus, observed heterozygosity (Ho), expected heterozygosity (He), probability of exclusion (PE), polymorphic information content (PIC), and inbreeding coefficient (Fis) were calculated using Parfex (version 1.0) [17], Cervus (version 3.0.7) [18], and Genepop (version 4.2) [19].

## 3. Results

### 3.1. Genetic Diversity

Comparative analysis of the SNP and STR markers revealed that the mean He ranged from 0.468 (AM) to 0.491 (JH) for SNP markers and from 0.695 (TH) to 0.791 (MH) for STR markers. The mean Ho ranged from 0.415 (AM) to 0.487 (MH) for SNP markers and from 0.706 (JH) to 0.776 (MH) for STR markers. The mean PIC ranged from 0.349 (AM) to 0.364 (MH) for SNP markers and from 0.635 (TB) to 0.761 (MH) for STR markers. The Fis ranged from −0.009 (MH) to 0.113 (AM) for SNP markers and from −0.058 (TB) to 0.043 (AM) for STR markers (Table 1 and Table 2).

The PE for the 71-SNPs panel exceeded 0.9999, indicating that the SNP markers may be sufficient for parentage testing. In comparison, the combined PE using STR markers was 0.9988 when one parent was known and 0.9999 when both parents were known.

### 3.2. Comparison of Parentage Testing Using STRs and SNPs

Paternity testing results using STR and SNP markers for each foal in the TB and MH breeds are shown in Table 3 and Table 4. For STR markers, paternity was not established in TBs owing to inconsistencies with Mendel’s laws of inheritance in five loci (AHT5, ASB17, ASB23, CA425, and LEX3). Similarly, in MHs, four loci (AHT4, ASB2, ASB17, and HMS2) displayed inconsistencies that led to paternity exclusion (Table 3).

Among the 71 SNP markers, 21 including MNEc_2_10_58909591_BIEC2_126732 in TBs and 27 including MNEc_2_31_17012751_BIEC2_839012 in MHs were inconsistent with Mendel’s laws of inheritance during parentage testing (Table 4).

## 4. Discussion

In the analysis of horse STR or SNP markers, the Fis value represents the inbreeding coefficient of an individual relative to the subpopulation, which is a population genetics parameter and one of Wright’s F-statistics (F-coefficients). In other words, it indicates the extent to which the observed heterozygosity within a subpopulation falls short of the expected heterozygosity. In this study, the negative Fis values observed in TBs and MHs suggest an excess of heterozygosity compared to expectations, reflecting rich genetic diversity. This can be indirectly inferred to result from outbreeding or selection rather than inbreeding. Also, Aminou et al. [4] reported that in Moroccan horses, the mean expected heterozygosity was 0.457 for SNPs and 0.76 for STRs, while the mean observed heterozygosity stood at 0.472 for SNPs and 0.72 for STRs. These findings were similar to the results of the present study, in which the mean He ranged from 0.468 (AM) to 0.491 (JH) for SNP markers and from 0.695 (TH) to 0.791 (MH) for STR markers, while the mean Ho ranged from 0.415 (AM) to 0.487 (MH) for SNP markers and from 0.706 (JH) to 0.776 (MH) for STR markers.

STRs are widely used in molecular analyses for individual identification and parentage verification in animals. Simple Mendelian inheritance patterns between offspring and candidate parents have been observed across many species [20,21,22,23]. In horses, ISAG regularly conducts comparative tests to ensure that laboratories performing DNA-based analyses maintain high standards of accuracy and consistency.

Efficient equine lineage registration requires the systematic processing of large sample sets and accurate allele identification. However, STR genotyping is not fully automated, and artifacts such as stutter peaks are common [5]. To address these issues, it is essential to recognize patterns of error and noise. Therefore, experienced professionals must manually verify automated genotype calls. In the case of STRs, ISAG recommends using as a minimum a twelve-marker panel for parentage testing, and if mismatches are observed in at least two markers, the case should be concluded as parentage exclusion. Accordingly, in this study, four to five markers showed mismatches, leading to a decision of parentage exclusion. This confirmed compliance with ISAG’s recommendations.

The transition from STR to SNP markers for livestock registration has gained increasing attention [24,25,26,27,28] and is currently underway for many livestock species [7,26,28]. SNPs offer several advantages over STRs, including greater genomic abundance and lower mutation rates [29]. Moreover, advances in sequencing technologies and reduced costs have made SNP-based analyses increasingly feasible [30]. Unlike STR genotyping, SNP genotyping is well suited to robotic automation, thereby improving testing efficiency [31].

Millions of SNP markers have been identified in horses [32,33]. Based on these SNP markers, Hirota et al. [5] and Holl et al. [25] developed lineage testing panels. Following their reports, ISAG conducted three rounds of SNP comparison tests in horses. Several SNP genotyping methods have been applied in horses, including MassARRAY [5], microarrays [25], and genotyping-by-sequencing [34]. Holl et al. [25] and Flynn et al. [34] evaluated the effectiveness of SNPs for parentage testing, prompting ISAG to further assess horse SNP panels through comparative studies.

Lee et al. [15] also reported the effectiveness of SNP markers for equine parentage testing. However, in Korea, parentage verification for registration purposes in TBs and JHs currently relies exclusively on STR markers. This study established an SNP-based parentage verification system for several horse breeds, including TBs, and evaluated its performance in comparison with the existing STR-based system.

Currently, ISAG recommends that when using SNPs for parentage verification, if the offspring and parent genotypes are opposing for a homozygous SNP, parentage is considered excluded when more than 10 mismatched markers are observed. In addition, if the offspring is heterozygous and both parents share the same homozygous genotype, parentage is considered excluded when more than 14 mismatched markers are observed. In this study, 21 to 27 mismatches were identified among the 71 SNP markers, indicating compliance with ISAG’s recommendations.

In Mongolia, horse pedigree registration is becoming increasingly important. Mongolia’s five major livestock species include sheep, goats, cattle, pigs, and horses [35], with MHs being particularly important. However, interbreeding between native MHs and improved breeds has raised concerns about preserving genetic purity, especially in horse racing. Therefore, establishing a comprehensive system for horse pedigree registration is urgently needed.

This study represents the first report in Korea of parentage testing in MHs using a large number of SNP markers. Our proposed approach may serve as a foundation for improving and developing the current parentage testing framework. The findings provide preliminary evidence that SNP markers can replace STR markers for horse parentage verification. As ISAG is currently conducting studies on SNP techniques across various horse breeds, this study also analyzed horses from five breeds. However, further studies involving larger sample sizes are required to validate these results.

SNP markers have been used to determine genetic relationships among horse populations [36], evaluate genetic diversity [37], and identify selection signatures [38]. In this study, genetic relationships among five horse populations were estimated using data from 15 STR loci. Among all populations, MHs exhibited the highest average heterozygosity (0.791). The cumulative exclusion probability (PE) for the 71-SNP panel exceeded 0.9999, indicating that SNP markers may be sufficient for parentage testing. In comparison, STR-based testing yielded a combined PE of 0.9988 when one parent was known and 0.9999 when both parents were known. Aminou et al. [4] compared the effectiveness of SNP markers and microsatellite markers (STRs) in parentage testing of Moroccan horses, reporting that SNP markers achieved a cumulative exclusion probability exceeding 99.99%, while microsatellite markers showed exclusion probabilities ranging from 99.8% to 99.9%. These findings suggest the high potential of SNPs for parentage verification, although further optimization of marker locus selection is needed. Similar results were observed in this study, aligning with the findings of Aminou et al. [4].

STR markers offer several advantages in parentage testing, including high polymorphism, accuracy, and rapid analysis. However, their limitations include a relatively small number of available loci and susceptibility to gene mutations. In the future, SNP technology is expected to enhance analytical resolution and accuracy while providing more comprehensive genetic information. This advancement may offer a stronger technical foundation for livestock breeding and the conservation of genetic resources [39].

This study demonstrated the potential of SNP testing as a foundational tool to improve and develop the current parentage verification system for horses in Korea and Mongolia. Although ISAG has not yet established a minimum set of SNP markers for horse parentage testing—unlike the defined minimum set for STRs—this study proposes a basic panel that could serve as a foundation in preparation for a possible future transition to SNP-based parentage testing by ISBC. Nevertheless, further research involving a larger number of samples and additional markers is necessary.

## 5. Conclusions

This study demonstrates the potential of SNP markers for parentage verification and individual identification in horses. SNP-based parentage testing could considerably enhance conservation efforts and breed management. This study proposes a basic panel that could serve as a foundation in preparation for a possible future transition to SNP-based parentage testing by ISBC. However, further research with larger sample sizes is required to confirm these findings and improve the reliability of SNP-based parentage testing systems.

## Figures and Tables

**Table 1:** Observed heterozygosity (Ho), expected heterozygosity (He), inbreeding coefficient (Fis), and polymorphic information content (PIC) for each locus and across different horse breeds based on short tandem repeat loci.

Marker	Ho					He				
	TB *	JH	QH	AM	MH	TB	JH	QH	AM	MH
**AHT4**	0.737	0.824	0.500	0.762	0.850	0.744	0.849	0.659	0.764	0.845
**AHT5**	0.868	0.824	0.700	0.714	0.817	0.690	0.754	0.775	0.764	0.788
**ASB2**	0.816	0.529	0.900	0.667	0.850	0.790	0.620	0.850	0.811	0.836
**ASB17**	0.816	0.882	0.700	0.810	0.893	0.761	0.803	0.829	0.832	0.889
**ASB23**	0.632	0.647	0.800	0.810	0.785	0.710	0.651	0.850	0.818	0.835
**CA425**	0.816	0.765	0.850	1.000	0.763	0.730	0.756	0.722	0.817	0.767
**HMS1**	0.605	0.588	0.700	0.571	0.634	0.568	0.586	0.633	0.638	0.708
**HMS2**	0.684	0.588	0.800	0.619	0.828	0.581	0.757	0.811	0.796	0.817
**HMS3**	0.763	0.882	0.950	0.667	0.613	0.620	0.822	0.826	0.768	0.783
**HMS6**	0.553	0.647	0.750	0.857	0.828	0.618	0.706	0.745	0.806	0.779
**HMS7**	0.868	0.412	0.650	0.857	0.613	0.767	0.438	0.818	0.835	0.606
**HTG4**	0.658	0.647	0.550	0.333	0.613	0.565	0.684	0.701	0.376	0.642
**HTG10**	0.763	0.882	0.850	0.762	0.839	0.751	0.781	0.825	0.842	0.866
**LEX3**	0.842	0.529	0.900	0.762	0.871	0.805	0.781	0.867	0.862	0.850
**VHL20**	0.605	0.941	0.850	0.905	0.850	0.721	0.748	0.855	0.862	0.849
**Mean**	0.735	0.706	0.763	0.740	0.776	0.695	0.716	0.784	0.773	0.791
**Marker**	**Fis**					**PIC**				
	**TB**	**JH**	**QH**	**AM**	**MH**	**TB**	**JH**	**QH**	**AM**	**MH**
**AHT4**	0.009	0.030	0.242	0.003	−0.006	0.685	0.802	0.601	0.708	0.821
**AHT5**	−0.259	−0.093	0.097	0.065	−0.038	0.636	0.702	0.714	0.715	0.751
**ASB2**	−0.033	0.145	−0.059	0.178	−0.016	0.748	0.559	0.808	0.758	0.810
**ASB17**	−0.072	−0.098	0.156	0.027	−0.004	0.711	0.753	0.781	0.788	0.874
**ASB23**	0.111	0.006	0.059	0.010	0.060	0.667	0.594	0.803	0.770	0.811
**CA425**	−0.117	−0.012	−0.177	−0.225	0.004	0.678	0.695	0.666	0.780	0.732
**HMS1**	−0.066	−0.003	−0.106	0.105	0.104	0.469	0.508	0.551	0.573	0.656
**HMS2**	−0.177	0.223	0.013	0.223	−0.014	0.507	0.685	0.767	0.740	0.785
**HMS3**	−0.231	−0.074	−0.150	0.132	0.217	0.578	0.770	0.782	0.704	0.749
**HMS6**	0.106	0.083	−0.007	−0.064	−0.063	0.535	0.629	0.688	0.755	0.740
**HMS7**	−0.133	0.059	0.206	−0.027	−0.012	0.720	0.385	0.763	0.789	0.572
**HTG4**	−0.165	0.054	0.216	0.114	0.045	0.462	0.609	0.622	0.346	0.606
**HTG10**	−0.017	−0.129	−0.030	0.095	0.031	0.702	0.724	0.782	0.798	0.846
**LEX3**	−0.046	0.364	−0.038	0.116	−0.025	0.767	0.771	0.828	0.819	0.827
**VHL20**	0.161	−0.258	−0.052	−0.050	−0.001	0.657	0.687	0.815	0.826	0.827
**Mean**	−0.062	0.020	0.025	0.047	0.019	0.635	0.658	0.731	0.725	0.761

* TB, Thoroughbred; JH, Jeju horse; QH, Quarter horse; AM, American Miniature; MH, Mongolian horse.

**Table 2:** Mean observed heterozygosity (Ho), expected heterozygosity (He), inbreeding coefficient (Fis), and polymorphic information content (PIC) across different horse breeds using STRs and SNPs.

Makers	Breeds	No. of Horses	No. of Markers	Ho	He	Fis	PIC
**STRs ** *	TB **	38	15	0.735	0.695	−0.058	0.635
	JH	17		0.706	0.719	0.018	0.658
	QH	20		0.767	0.785	0.023	0.731
	AM	21		0.740	0.773	0.043	0.725
	MH	93		0.776	0.791	0.018	0.761
**SNPs**	TB	38	71	0.451	0.471	0.041	0.355
	JH	17		0.470	0.491	0.043	0.362
	QH	20		0.460	0.477	0.036	0.355
	AM	18		0.415	0.468	0.113	0.349
	MH	93		0.487	0.483	−0.009	0.364

* STRs, short tandem repeats or microsatellites; SNPs, single-nucleotide polymorphisms. ** TB, Thoroughbred; JH, Jeju horse; QH, Quarter horse; AM, American Miniature; MH, Mongolian horse.

**Table 3:** Cases of paternity exclusion in horse parentage testing using short tandem repeats.

	Marker															Breeds
	AHT4	AHT5	ASB2	HMS3	HMS6	HMS7	HTG4	HTG10	VHL20	ASB17	ASB23	HMS1	LEX3	CA425	HMS2	
**Sire**	H/O	K/M	M/Q	I/O	P/P	M/N	K/M	I/I	L/M	N/O	K/K	J/M	M/-	N/O	K/L	**TB ** **
**Dam**	J/O	J/K	N/R	I/O	P/P	O/O	K/K	I/R	I/I	N/R	J/S	J/M	H/O	J/N	K/L	
**Foal**	O/O	**M/N ** *	M/N	I/I	P/P	N/O	K/M	I/I	I/L	**M/R**	**L/S**	J/M	**N/-**	**M/N**	L/L	
**Sire**	I/O	J/N	K/M	I/N	L/P	N/O	P/P	L/S	I/M	M/Q	J/U	M/M	O/O	J/M	M/O	**MH**
**Dam**	H/O	N/N	K/K	I/I	L/O	O/O	K/P	R/S	I/Q	N/Q	L/U	J/M	L/O	J/N	J/O	
**Foal**	**J/O**	J/N	**K/N**	I/I	L/L	O/O	K/P	L/S	M/Q	**M/M**	U/U	M/M	O/O	J/N	**J/N**	

* Bold and underlined text indicates alleles that are inconsistent with Mendel’s law of inheritance. ** TB, Thoroughbred; MH, Mongolian horse.

**Table 4:** Cases of paternity exclusion in horse parentage testing using single-nucleotide polymorphisms.

Marker	TB *			MH		
	Sire	Dam	Foal	Sire	Dam	Foal
**MNEc_2_10_58909591_BIEC2_126732**	AB **	BB	**AA ** ***	AB	AB	AA
**MNEc_2_6_31320852_BIEC2_946446**	AB	AA	AA	AB	BB	AB
**MNEc_2_16_81464884_BIEC2_364741**	BB	BB	**AB**	AA	AA	**AB**
**MNEc_2_10_43452669_BIEC2_119640**	AA	AB	AA	AB	AB	BB
**MNEc_2_31_17012751_BIEC2_839012**	AB	BB	AB	AB	BB	**AA**
**MNEc_2_26_29137373_BIEC2_692543**	AB	BB	AB	AB	AB	AB
**MNEc_2_8_61558651_BIEC2_1057053**	BB	AB	AB	BB	BB	**AB**

* TB, Thoroughbred; MH, Mongolian horse. ** SNP bases A and T were classified as allele A, and C and G were classified as allele B. *** Bold and underlined text indicates alleles that are inconsistent with Mendel’s laws of inheritance.

## Data Availability

All data supporting the findings of this study are available from the corresponding author upon request.
